# Epidemiology of *Cryptococcus gattii*, British Columbia, Canada, 1999–2007

**DOI:** 10.3201/eid1602.090900

**Published:** 2010-02

**Authors:** Eleni Galanis, Laura MacDougall, Sarah Kidd, Mohammad Morshed, the British Columbia Cryptococcus gattii Working Group

**Affiliations:** British Columbia Centre for Disease Control, Vancouver, British Columbia, Canada (E. Galanis, L. MacDougall); University of British Columbia, Vancouver (E. Galanis)

**Keywords:** Cryptococcus gattii, fungus epidemiology, fungi, hospitalization, mortality, British Columbia, Canada, research

## Abstract

British Columbia, Canada, has the largest reported population of *Cryptococcus gattii*–infected persons worldwide. To assess the impact of illness, we retrospectively analyzed demographic and clinical features of reported cases, hospitalizations, and deaths during 1999–2007. A total of 218 cases were reported (average annual incidence 5.8 per million persons). Most persons who sought treatment had respiratory illness (76.6%) or lung cryptococcoma (75.4%). Persons without HIV/AIDS hospitalized with cryptococcosis were more likely than those with HIV/AIDS to be older and admitted for pulmonary cryptococcosis. The 19 (8.7%) persons who died were more likely to be older and to have central nervous system disease and infection from the VGIIb strain. Although incidence in British Columbia is high, the predominant strain (VGIIa) does not seem to cause greater illness or death than do other strains. Further studies are needed to explain host and strain characteristics for regional differences in populations affected and disease outcomes.

## Abstract 2

Incidence is high, but the predominant strain does not seem to cause greater illness or death than do other strains.

*Cryptoccocus gattii* is an environmental fungus that emerged in a temperate climate on Vancouver Island, British Columbia, Canada, in 1999, causing an outbreak affecting humans and animals (*1*,*2*). Previously, *C. gattii* had been reported only from primarily tropical and subtropical regions (*3*,*4*); since then, >200 human cases have been documented in British Columbia.

In British Columbia, *C. gattii* colonizes various species of trees and soil and has been recovered from water and air (*5*). Humans become infected by inhaling yeasts or spores. The primary site of infection is the lung; *C. gattii* can lead to pneumonia or formation of cryptococcomas. The infection can disseminate to most other organs, notably the central nervous system (CNS), where it causes meningoencephalitis or brain cryptococcomas (*6*,*7*). Infection is thought to occur mostly in immunocompetent persons, but new evidence from British Columbia shows that a sizeable proportion of persons with *C. gattii* have underlying immunocompromising conditions (E. Galanis, L. MacDougall, unpub. data). This contrasts with *C. neoformans*, which is distributed worldwide and causes mostly CNS infections in immunocompromised persons, particularly HIV-infected persons (*6*,*7*).

Some researchers have suggested that VGIIa, the predominant strain in British Columbia, is more virulent than other strains found in other countries (*4*,*8*,*9*). Most studies of epidemiologic and clinical aspects of *C. gattii* have shown fewer illnesses and deaths from *C. gattii* infection than from *C. neoformans* (*10*,*11*); however, some have shown they are higher (*12*).

British Columbia has the largest documented population of *C. gattii*–infected persons worldwide. To clarify the epidemiology and impact of illness caused by *C. gattii* infection, we retrospectively analyzed incidence of, hospitalizations for, and deaths caused by *C. gattii* in British Columbia from its emergence in 1999 through 2007.

## Methods

Incidence was derived from cases reported to public health authorities. Because complete information about reported cases was not available, hospitalization and death rates were derived from administrative registries.

### Case Definitions

BC laboratories report persons infected with *Cryptococcus* spp. to public health authorities (population-based surveillance). We analyzed *C. gattii* infection diagnosed during 1999–2007 in BC residents and reported to the BC Centre for Disease Control. A confirmed case was defined as culture-confirmed *C. gattii* infection based on differential media and genotyping (*13*). A probable case was defined as laboratory evidence of infection from antigen detection, histopathology, or microscopy in an HIV-negative person. For their infection to be considered a case, patients must have traveled to or resided in a local *C. gattii*–endemic area during the year before onset. Local *C. gattii*–endemic areas were Vancouver Island since 1999 and the greater Vancouver area and Fraser Valley of the BC mainland since 2004 (*13*). We defined a case acquired on the BC mainland as a confirmed case in a person who did not travel to Vancouver Island or to any international *C. gattii*–endemic area during the year before illness onset.

Case-patients were classified as having a respiratory syndrome if they reported cough or an abnormal chest radiograph or had microbiologic evidence of *Cryptoccocus* in a respiratory specimen. Case-patients were classified as having a CNS syndrome if they had abnormal brain imaging or microbiologic evidence of *Cryptococcus* in a brain or cerebrospinal fluid specimen. Case-patients with other presentations had microbiologic evidence of *Cryptococcus* in another organ/tissue. Case-patients who were HIV positive or had a history of invasive cancer or organ transplant or were on corticosteroids in the year before onset were considered immunocompromised.

### Incidence, Epidemiology, and Clinical Characteristics

Incidence, epidemiology, and clinical characteristics were analyzed for confirmed, probable, and all cases and compared between confirmed and probable cases. We derived incidence using annual BC population estimates. We calculated frequencies using the case-patients from whom the information was available, which varied for each question or data point.

### Hospitalizations

Hospital discharge data for International Classification of Diseases, Ninth Revision (ICD-9), code 117.5 and Tenth Revision (ICD-10) code B45.X (B45.0, B45.1, B45.2, B45.3, B45.7, B45.8, B45.9) were obtained from the BC Hospital Separations/Discharge Abstract Database, which captures all hospital visits in the province, for 1999–2006 (*14*). (British Columbia switched from ICD-9 to ICD-10 coding in 2001.) Records with the same unique identifier and age (adjusted for date of hospitalization) were considered to represent the same person because unique identifiers for certain types of patients were recycled. Annual hospitalization rates were derived by adding the earliest hospitalization for each individual and dividing by the annual BC population. Additional hospitalizations for the same person were considered repeat hospitalizations.

Because ICD-9 and ICD-10 have no *C. gattii*-specific code, we analyzed persons hospitalized with cryptococcosis without HIV/AIDS. We chose this population as a proxy for *C. gattii* infection because its hospitalization rate increased sharply in 1999, signaling onset of the outbreak (*1*). In addition, few (6.2%) confirmed *C. gattii*–infected persons have HIV infection in British Columbia (E. Galanis, L. MacDougall, unpub. data). HIV/AIDS was defined as ICD-9 codes V08 and 042.X (42.0, 42.1, 42.2, 42.8, 42.9) and ICD-10 codes B20, B21, B22, B23, and B24. Cases of cryptococcosis were separated into those with no ICD code for HIV/AIDS reported on any hospitalization record within the study period and those with an ICD code for HIV/AIDS reported at least once. Among cases with an ICD-10 code for cryptococcosis, subcodes were analyzed. (Subcodes for cryptococcosis are not available in ICD-9.)

### Deaths

Non-nominal data for deaths from cryptoccocal infection were obtained from BC Vital Statistics, which includes all deaths among BC residents (*15*). All deaths occurring during 1999–2007 for which the underlying or a contributing cause of death was noted as ICD-9 code 117.5 or ICD-10 code B45.X were extracted. We identified deaths in persons with *C. gattii* infection by matching birth date with cases reported to the BC Centre for Disease Control. We derived annual death rates by dividing the number of deaths by the annual BC population.

### Population Data, Analysis and Software Used, and Patient Consent

Population data were obtained from British Columbia Statistics (*16*). Using the χ^2^ test and Fisher exact test, we compared independent proportions; the t test, to compare means; and the Mann-Whitney test, to compare non-normally distributed results. We used the Bonferroni correction to compare strain distribution by age group. Data were analyzed by using SPSS v16.0 (SPSS Inc., Chicago, IL, USA) and StatXact-6 v6.2.0. (StataCorp LP, College Station, TX, USA) Because the data were obtained from surveillance and administrative sources and presented in aggregate format, patient consent was not obtained.

## Results

### Incidence, Epidemiology, and Clinical Characteristics

**Figure 1:**
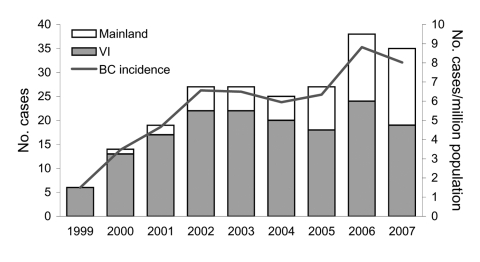
Number of cases of *Cryptococcus gattii* infection and incidence rate per million population, by case-patient place of residence, British Columbia (BC), Canada, 1999–2007. Mainland, mainland BC; VI, Vancouver Island.

A total of 218 cases (124 confirmed and 94 probable) of *C. gattii* infection were reported during 1999–2007 (Table 1). An average of 24.2 cases was reported every year (5.8/million/year); cases increased steadily from 6 in 1999 to 38 in 2006 (Figure 1). Onset did not vary by season or by month. Nearly three quarters (73.9%) of all case-patients lived on Vancouver Island (average annual incidence rate 25.1/million). The number of cases reported per year reached a plateau on Vancouver Island in 2002 but has increased on the mainland since 2005. Seven confirmed cases were acquired on the BC mainland (1 in 2004, 2 in 2005, 3 in 2006, and 1 in 2007).

**Table 1:** Characteristics of persons with cases of confirmed or probable *Cryptococcus gattii* infection, British Columbia, Canada, 1999–2007*

Characteristic	Total	Confirmed	Probable	p value
No. cases	218	124	94	NA
Average incidence (million/year)	5.8	3.3	2.5	NA
No. cases in persons living on Vancouver Island	161 (73.9)	83 (66.9)	78 (83.0)	0.177
Demographic data				
Male sex	121 (55.5)	73 (58.9)	48 (51.1)	0.440
Age, y				
Mean	58.7	58.7	58.7	0.988
Range	2–92	2–92	12–87	NA
Children <18 y	4 (1.8)	3 (2.4)	1 (1.1)	0.463
Clinical assessment				
Respiratory syndrome	167 (76.6)	85 (68.5)	82 (87.2)	0.031
CNS syndrome	17 (7.8)	16 (12.9)	1 (1.1)	0.001
Respiratory and CNS syndrome	22 (10.1)	20 (16.1)	2 (2.1)	<0.001
Other/unknown	12 (5.5)	3 (2.4)	9 (9.6)	NA
Asymptomatic	16 (7.3)	6 (4.8)	10 (11.0)	0.120
Hospitalized	98 (60.9)	58 (65.2)	40 (55.6)	0.434
Immunocompromised	70 (38.0)	41 (38.7)	29 (37.2)	0.870

*Frequencies were calculated by using persons from whom information was available, which varied for each question or data point. All values given as no. (%) persons except as indicated. NA, not available; CNS, central nervous system.

Just over half (55.5%) of all case-patients were male. The mean age was 58.7 years. The incidence rate increased with age, with the highest age-specific rate in persons 70–79 years of age.

A total of 167 (76.6%) case-patients sought treatment for a respiratory syndrome; 17 (7.8%), for a CNS syndrome; 22 (10.1%), for both respiratory and CNS syndromes; and 1 each, for a combination of skin and respiratory, skin and CNS, and sepsis and respiratory syndrome (Table 1). The most common symptoms reported in case-patients with a respiratory syndrome were cough, dyspnea, and chest pain. Case-patients with a CNS syndrome most commonly reported headache, night sweats, weight loss, anorexia, and neck stiffness. Eighty-nine (75.4%) of the118 case-patients who had abnormal chest radiographs had single or multiple lung nodules. Sixteen (7.3%) cases were asymptomatic. All had a respiratory syndrome, and all who reported chest radiograph results had evidence of single or multiple lung nodules. Age, sex, and genotype did not differ significantly from those of symptomatic cases. Ninety-eight (60.9%) case-patients were hospitalized. Seventy (38.0%) case-patients were considered immunocompromised; 81.4% had a respiratory syndrome and 5.7% had CNS signs. Being immunocompromised was not associated with clinical presentation (p = 0.385).

Patients with confirmed and probable cases did not differ significantly by age and sex or proportions residing on Vancouver Island, asymptomatic, hospitalized, or immunocompromised (Table 1). Persons with confirmed cases were more likely than persons with probable cases to have a CNS syndrome only (p = 0.001) or a CNS and respiratory syndrome (p<0.001) and less likely to have a respiratory syndrome only (p = 0.031).

Three confirmed cases occurred in children 2, 5, and 16 years of age. Each had a respiratory syndrome; 1 was asymptomatic. All were HIV negative. Two were on inhaled corticosteroids. One had a chronic respiratory disease, 1 had a genetic disorder; the third was otherwise well.

**Figure 2:**
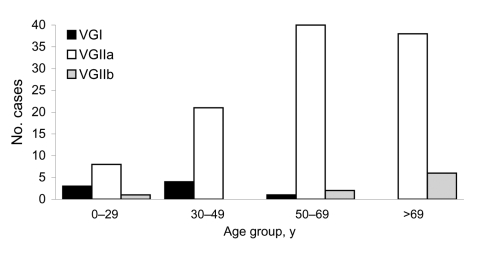
Distribution of *Cryptococcus gattii* strains among 124 persons with *C. gattii* infection, by age category, British Columbia, Canada, 1999–2007.

The VGIIa strain was responsible for 107 (86.3%) of confirmed cases. Eight case-patients were infected with VGI and 9 with VGIIb. Clinical presentation did not differ significantly by genotype. However, strains differed by patient age; case-patients >50 years of age were more likely to be infected with VGIIa (p = 0.002) or VGIIb (p = 0.006) than with VGI (Figure 2).

### Hospitalizations

**Figure 3:**
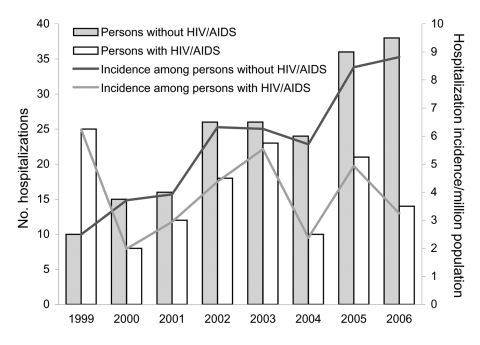
Comparison of hospitalizations for cryptococcosis among persons with and without HIV/AIDS, British Columbia, Canada, 1999–2006.

During 1999–2006, a total of 322 persons were hospitalized with cryptococcosis, for a total of 533 hospitalizations. A total of 191 (59.3%) persons did not have HIV/AIDS. An average of 21.2 persons without HIV/AIDS were hospitalized for cryptococcosis each year; this number increased steadily from 10 in 1999 to 38 in 2006 (Figure 3). At 5.8 hospitalizations per million BC residents, the average annual hospitalization rate was higher for cryptococcosis without HIV/AIDS than with HIV/AIDS (p = 0.004) (Table 2). Persons with HIV/AIDS had more admissions per person and were hospitalized for longer periods (both p<0.001). Hospitalized persons without HIV/AIDS were less likely to be male (p = 0.007) and more likely to be older (p<0.001) than those with HIV/AIDS.

**Table 2:** Characteristics of hospitalizations and demographic data for 322 persons hospitalized for cryptococcosis, British Columbia, Canada, 1999–2006

Characteristic	Cryptococcosis without HIV/AIDS	Cryptococcosis with HIV/AIDS	p value
No. hospitalizations	266	267	
No. case-patients	191	131	
Incidence*	5.8	3.9	0.004
No. admissions per case-patient, median (range)	1 (1–7)	3 (1–11)	<0.001
Length of stay, d, median (range)	8 (0–142)	14 (1–288)	<0.001
Demographic data			
Male sex, %	56.5	82.4	0.007
Age, y			
Mean	57.9	40.3	<0.001
Range	1–91	6–75	

*Average annual hospitalization rate per million British Columbia population.

Among cryptococcosis hospitalizations for which an ICD-10 code was available, most (56.5%) hospitalizations for persons without HIV/AIDS were for pulmonary cryptococcosis; most (77.7%) hospitalizations for persons with HIV/AIDS were for cerebral cryptococcosis (Table 3). Persons without HIV/AIDS were more likely to be hospitalized with pulmonary (p<0.001) or cutaneous (p = 0.044) disease but less likely to be hospitalized with cerebral (p<0.001) or disseminated (p<0.001) disease than were persons with HIV/AIDS.

**Table 3:** ICD-10 codes for cryptococcosis hospitalizations, British Columbia, Canada, 1999–2006 (N = 322)*

Condition (ICD-10 code)	Cryptococcosis without HIV/AIDS, no. (%)†	Cryptococcosis with HIV/AIDS, no. (%)†	p value
Pulmonary cryptococcosis (B45.0)	130 (56.5)	13 (6.7)	<0.001
Cerebral cryptococcosis (B45.1)	63 (27.4)	150 (77.7)	<0.001
Cutaneous cryptococcosis (B45.2)	7 (3.0)	1 (0.5)	0.044
Osseous cryptococcosis (B45.3)	1 (0.4)	0	ND
Disseminated cryptococcosis (B45.7)	5 (2.2)	22 (11.4)	<0.001
Other forms of cryptococcosis (B45.8)	11 (4.8)	6 (3.1)	0.289
Cryptococcus, unspecified (B45.9)	13 (5.7)	12 (6.2)	0.834
Missing ICD-10 code	36	74	
Total	266	267	

*ICD-10, International Classification of Diseases, tenth revision; ND, not determined. †Total hospitalizations minus hospitalizations for which ICD code is missing.

### Deaths

During 1999–2007, 19 case-patients (case-fatality ratio [CFR] 8.7%) died from or with *C. gattii* infection, of which 15 cases were confirmed (CFR = 12.1%). The *C. gattii*–specific average annual death rate, based on all deaths, was 0.5/million. During the same period, 42 case-patients died with cryptococcosis and HIV/AIDS infection, for an average annual death rate of 1.1/million in British Columbia.

Thirteen (68.4%) case-patients who died were male, but this percentage did not significantly differ from that of case-patients who survived (p = 0.474). Mean age at death was 67.6 years (range 26–91 years). Persons who died were older at diagnosis than survivors (p = 0.019) and more likely to have CNS syndrome with or without respiratory syndrome (p = 0.014). Ten (66.7%) persons with confirmed cases who died had an infection caused by VGIIa; 4 (26.7%), by VGIIb; and 1 (6.7%), by VGI. Case-patients who died were more likely to have been infected by VGIIb than by the other 2 strains combined (p = 0.002).

On the basis of public health interviews and death certificates, 14 (73.7%) of all case-patients who died had underlying medical conditions that might have increased their risk for death, including cancer, chronic obstructive pulmonary disease, asthma, liver disease, diabetes, HIV infection, lung transplant, congestive heart failure, and congenital heart malformation. Nine (47.4%) were immunocompromised; case-patients who died were not more likely than survivors to be immunocompromised (p = 0.267).

## Discussion

We have presented a population-based assessment of the epidemiology and impact of illness of *C. gattii* infection in a newly endemic part of the world. Although a number of studies have assessed epidemiologic and clinical features of cryptococcosis, few have done so for *C. gattii* cases separately, particularly in recent years and outside Australia (*10*–*12*,*17*). Because few regions other than British Columbia and France have ongoing population-based surveillance for cryptococcosis, most previous studies were based on retrospective hospital chart reviews or surveys (*18*).

The average annual incidence of *C. gattii* infection on Vancouver Island (25.1/million) is one of the highest in the world. Australia reported an annual cryptococcal infection incidence of 140/million in Aboriginals in Arnhemland, Northern Territory, in 1976–1992 (*19*); a total of 77.8% of these cases were caused by *C. gattii*. Papua New Guinea reported an annual *C. gattii* incidence of 42.8/million in the Central Province in 1993–1995, but this included cases in residents of other provinces (*20*). The reason for the high incidence in British Columbia and these other regions is unclear but may be due to ecologic, host, or strain-related characteristics.

The incidence in British Columbia increased in the early years of the emergence (1999–2001), either because of increasing awareness and reporting or a true increase in incidence from increased fungal concentration over time or gradual cumulative infection of exposed susceptible persons. The incidence on Vancouver Island stabilized in 2002, but the overall BC incidence increased in 2006–2007 because of an increased number of case-patients residing on the BC mainland, suggesting that the true number of persons exposed there may be higher than we estimated using our specific case definition for acquisition on the mainland.

Although the definition for a confirmed case is more specific, all variables analyzed are similar for confirmed and probable cases except clinical presentation. Cases that include a CNS syndrome may be more likely to be confirmed through a cerebrospinal fluid culture than are cases that include respiratory syndrome, which require a more invasive pulmonary sample for culture confirmation.

*C. gattii* incidence rates and trends in British Columbia are similar to the cryptococcosis hospitalization rate for persons without HIV/AIDS. However, only 60.9% of *C. gattii*–infected case-patients reported being hospitalized for their illness. This discrepancy most likely results from hospitalization of persons with other immunocompromising conditions whose cryptococcal infection was caused by *C. neoformans* rather than *C. gattii*. Before *C. gattii* emerged in 1999, an average of 11 persons were hospitalized with cryptococcosis without HIV/AIDS in British Columbia each year (*1*). This average is similar to the annual incidence of cryptococcosis in persons without HIV/AIDS in non–*C. gattii*–endemic regions (*7*,*21*–*23*). Therefore, the true *C. gattii* hospitalization rate is probably lower than that reported here. Lack of a *C. gattii*–specific ICD code and exclusion of persons hospitalized before 1999 limited our assessment.

Most (90.8%) *C. gattii*–infected persons sought treatment for respiratory syndrome with or without neurologic findings. Respiratory presentations and pulmonary cryptococcomas are commonly associated with *C. gattii* or cryptococcal infections in immunocompetent patients (*10*,*24*). Only 18.3% of BC cases had evidence of CNS involvement initially, which contrasts with an Australian study of hospitalized patients in which 85% of 20 *C. gattii*–infected patients had meningitis and a nationwide survey in Colombia in which 93.3% of 30 had neurologic findings (*10*,*17*). Also in contrast to other authors, we found that case-patients without HIV/AIDS were admitted less often and hospitalized for less time than those with HIV/AIDS (*12*). These differences may be due to the timing of data collection in the progression of disease (which may have occurred earlier in British Columbia); the fact that the case-patients in the Australian studies were all hospitalized (and probably more seriously ill); and differences in study methods, medical practices, and strain characteristics. Our analysis was limited by the self-reported nature of the data, which lacked specificity and detail.

Published estimates of CFRs vary widely; from 0% of 20 cases to 15% of 26 cases with CNS disease in Australia (*10*,*12*). The CFR in British Columbia (8.7%) is probably a more stable estimate given the large number of case-patients for whom information was available and, possibly, the different strain distribution. BC case-patients who died were more likely to be older, have CNS disease, and be infected with VGIIb. The small numbers preclude assessment of whether death was independently associated with age or genotype. Other studies also have found that CNS disease and age increase the risk for death (*12*,*25*).

Of *C. gattii*-infected persons, 7.3% (9.6% of cases with a respiratory syndrome) were asymptomatic. The few studies describing this finding report that approximately one third of pulmonary cryptococcal cases are asymptomatic (*24*,*26*,*27*). The much lower proportion of asymptomatic cases in British Columbia may result from species or strain differences or diagnostic and reporting practices.

In British Columbia, 3 (2.4%) persons with confirmed cases were <18 years of age. In most *C. gattii*–endemic areas except northern Brazil, cryptococcosis in children is rarely reported (*12*,*17*,*28*–*30*). Two of the BC pediatric case-patients had underlying conditions affecting their lungs and were on inhaled corticosteroids. Diminished respiratory function or some level of immunocompromise may be necessary for children to become infected with *C. gattii*. The natural history of disease remains unclear; symptomatic disease may be associated with recent exposure, prolonged incubation, or reactivation of latent disease (*31*,*32*). In British Columbia, the incidence is highest in the 70–79-year age group, which may be linked to underlying medical conditions or to decreasing age-related cellular immunity, both of which could lead to acute infection or reactivation of latent disease.

Mouse models show that VGIIa may be more virulent than VGI and *C. neoformans* (*9*). Although *C. gattii* incidence is comparatively high in British Columbia, we could not find evidence that VGIIa causes more severe illness and a higher death rate than do other strains or *C. neoformans*. In British Columbia, persons hospitalized with cryptococcosis and HIV/AIDS (a proxy for *C. neoformans* infection) were more likely than those without HIV/AIDS to be hospitalized with severe disease. The death rate for case-patients with HIV/AIDS was twice as high as that for persons with *C. gattii* infection. In British Columbia, *C. gattii*–infected persons were less likely to present with CNS disease and no more likely to die from their infection than those in Australia, most of which is caused by VGI. In addition, we found that VGIIb infections may be more likely to be associated with a fatal outcome than VGIIa or VGI infections, either directly or because they affect older persons. This finding might be related to a strain-specific ability to reactivate. This association merits further study. To further assess the pathogenicity of *Cryptococcus* strains, common genotyping methods need to be used routinely to compare strains and outcomes internationally.

Evidence is increasing that *C. gattii* affects different populations and has a different clinical presentation and outcome than *C. neoformans* infection. Whether this is due to strain or host characteristics remains unclear. Speciation of *Cryptococcus* and laboratory-based surveillance should be considered for all areas where *C. gattii* is known to be, or is possibly, endemic. Where the disease is not reportable, a *C. gattii*–specific ICD code would allow surveillance of hospitalized case-patients. Raising awareness among physicians is necessary to ensure appropriate specimens are collected for culture and diagnoses are accurately reported.

Understanding of the progression of *C. gattii* infection and disease is lacking. *C. gattii*–specific serologic tools and long-term studies are needed to better understand natural progression and factors that impact outcome to better manage the risk associated with *C. gattii* and patients affected by it.

We have provided evidence of substantial *C. gattii*–related illness and of continued yet limited acquisition on the BC mainland since 2004. With its recent identification in the US Pacific Northwest, standardized laboratory-based surveillance and sharing of epidemiologic data are necessary to increase understanding of how and where this elusive pathogen spreads (*13*,*33*–*35*).
