# Errata—Vol. 16, No. 2

**DOI:** 10.3201/eid1604.C21604

**Published:** 2010-04

**Keywords:** errata, erratum, correction

The author list for the article Epidemiology of Cryptoccocus gattii, British Colombia, Canada, 1999–2007 (E. Galanis et al.) was incomplete. Authors were Eleni Galanis, Laura MacDougall, Sarah Kidd, Mohammad Morshed, and the British Columbia Cryptococcus gattii Working Group. Working Group members involved in this study were Patrick Doyle, John Galbraith, Linda Hoang, Pamela Kibsey, Min-Kuang Lee, Sultana Mithani, Marc Romney, and Diane Roscoe. The article has been corrected online http://wwwnc.cdc.gov/eid/article/16/2/09-0900_article.htm. 

