# Errata—Vol. 16, No. 2

**DOI:** 10.3201/eid1709.C11709

**Published:** 2011-09

Some data were listed incorrectly in Table 1 and the text in the article Epidemiology of *Cryptococcus gattii*, British Columbia, Canada, 1999–2007 (E. Galanis et al.). The article has been corrected online (www.cdc.gov/eid/content/16/2/251.htm).

